# A156 INCIDENCE OF MALIGNANT GASTRIC OUTLET OBSTRUCTION IN PATIENTS WITH PANCREATIC CANCER UNDERGOING FOLFIRINOX THERAPY

**DOI:** 10.1093/jcag/gwad061.156

**Published:** 2024-02-14

**Authors:** Y Rostamianmoghaddam, J Barkun, A Qatomah, C Long, Y Chen

**Affiliations:** Experimental Medicine, McGill University, Montreal, QC, Canada; Experimental Medicine, McGill University, Montreal, QC, Canada; Experimental Medicine, McGill University, Montreal, QC, Canada; Experimental Medicine, McGill University, Montreal, QC, Canada; Experimental Medicine, McGill University, Montreal, QC, Canada

## Abstract

**Background:**

Malignant gastric outlet obstruction is a morbid complication of pancreatic cancer. There is currently a lack of epidemiology data characterizing its incidence and outcomes in patients with unresectable pancreatic head cancer undergoing chemotherapy with leucovorin, fluorouracil, irinotecan, and oxaliplatin (FOLFIRINOX). Determining the incidence of MGOO in this context is crucial for developing effective management strategies.

**Aims:**

We aim to ascertain the incidence of MGOO in patients with pancreatic cancer treated with FOLFIRINOX. Secondary objectives include evaluating the clinical outcomes of MGOO. The primary endpoint was the rate of malignant gastric outlet obstruction, defined as clinical evidence of MGOO confirmed with axial imaging and/or endoscopy. Secondary endpoints included the length of hospitalization for MGOO and the rate of successful endoscopic or surgical management.

**Methods:**

This was a retrospective, single-center study including patients with pancreatic head cancer treated with FOLFIRINOX between January 1, 2017, and December 31, 2022, who did not undergo surgical resection. Patients were excluded if they had pancreatic cancer located in the neck, body, and/or tail of the pancreas, had predominantly cystic cancer, had an oncological resection, had a prophylactic surgical gastrojejunostomy, or had metastatic cancer to the pancreas.

**Results:**

Overall, 44 patients with pancreatic head cancer (40.90% female, mean age 59.3 ± 8.5 years) were included. There was no statistically significant difference in tumor staging between MGOO and No-MGOO patients: overall, 6.8% borderline, 40.9% metastatic, and 52.3% locally advanced. The incidence of MGOO in patients with pancreatic head cancer was 36.36%. Patients who never developed MGOO experienced significantly shorter unplanned hospitalizations (mean ± SD: 20.8 ± 18.30 days) compared to those with MGOO (mean ± SD: 50.33 ± 41.22 days; p = 0.0175). The mean number of FOLFIRINOX cycles received was 7.2 (±5.43) for patients without MGOO and 8.1 (±5.81) for patients with MGOO (p = 0.6152). Among patients with MGOO (n=12), management strategies included EUS-guided gastrojejunostomy (43.75%), duodenal stent placement (31.25%), and medical management (25%).

**Conclusions:**

Our contemporary data suggest a high rate of MGOO (36%) in patients with advanced pancreatic head cancer undergoing FOLFIRINOX treatment. The development of MGOO was associated with longer total hospitalization when compared to patients who never developed MGOO. Our study underscores the significant negative impact of MGOO with further research needed to elucidate better management strategies including better endoscopic or surgical prophylactic measures.

**Funding Agencies:**

CIHR

